# Case report: Acrodermatitis enteropathica result from a novel *SLC39A4* gene mutation

**DOI:** 10.3389/fped.2022.972030

**Published:** 2022-11-21

**Authors:** Wenjing Hua, Jialin Zou, Yuan Zhuang, Taiguang Zhou

**Affiliations:** ^1^ Department of Pediatrics, The Affiliated Hospital of Southwest Medical University, Luzhou, China; ^2^ Department of Emergency, West China Second University Hospital, Sichuan University, Chengdu, China

**Keywords:** acrodermatitis enteropathica, *SLC39A4* gene, mutation, zinc deficiency, child

## Abstract

The clinical data and gene sequencing results in a child with acrodermatitis enteropathica were retrospectively reported, and the related literature was reviewed. A girl aged 9 years and 4 months presented with a repeated skin rash, mainly distributed in the perioral, anogenital, and acral areas, accompanied with alopecia, and a low blood zinc level was found many times. A significant improvement was seen after continuous zinc supplementation. The genetic sequencing test demonstrated that the patient had compound heterozygous for two *SLC39A4* mutations: c.1466dupT (p.S490Efs*155) and c.295G > A (p.A99T), and her parents were heterozygous carriers of these two mutations. An improvement was achieved after continuous zinc supplementation. This case report might guide further research on this aspect.

## Introduction

Acrodermatitis enteropathica (AE) is a rare hereditary disorder due to zinc deficiency, characterized by the presence of dermatitis (mainly perioral, perianal, and acromelic), alopecia, diarrhea, growth restriction, and depressed mental function in early childhood. AE is an autosomal recessive disease that was first described by Danbolt and Closs (1) in 1942. In 2002, Kury et al. (2) identified the *SLC39A4* gene located in 8q24.3 as the pathogenic gene of AE. Mutations in the *SLC39A4* gene can cause zinc deficiency and rashes. *SLC39A4* mutations are found throughout the gene, including many different types of mutations. In this paper, we studied a family of AE and found a mutation in the *SLC39A4* gene, which has not been reported before, and reported as follows.

## Case report

**Figure 1:**
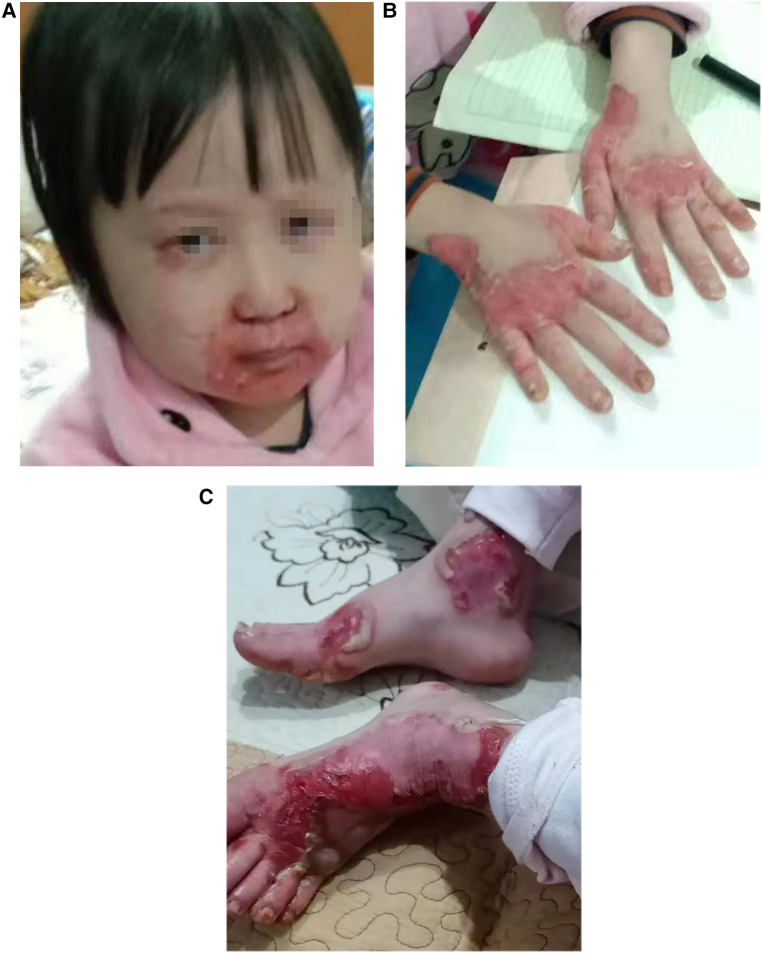
(**A–C**) Clinical photos of the AE patient before treatment: (**A**) the rashes over perioral area. (**B,C**) The rashes on hands and feet.

**Figure 2:**
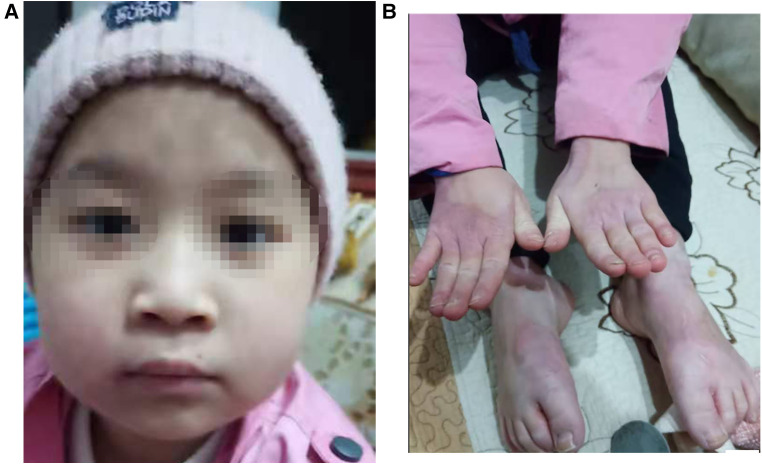
(**A,B**) Clinical photos of the AE patient after treatment: (**A**) the rashes over perioral area. (**B**) The rashes on hands and feet.

The patient is a girl aged 9 years and 4 months, admitted to the hospital due to “recurrent rash for 8 years and relapse for 1 month.” The rashes first occurred in the child when she was 1 year old. The rash was mainly perioral, perianal, and acromelic in distribution, accompanied with alopecia, and without diarrhea. The patient was taken to several hospitals because of “dermatitis,” and the lab test suggested zinc deficiency. After treatment with an oral zinc supplement, the patient’s rash improved without scarring or pigmentation but was easily repeated after stopping the zinc supplement for a period, often relapsed by the turn of autumn and winter. Recently, the patient has not taken zinc supplement regularly. The patient developed multiple skin rashes all over her body for almost 1 month, so she was admitted to our pediatric department for further evaluation. She had no allergic disorders. Family members included her parents, her two half-brothers through her mother and father, respectively, and her half-sister through her mother were in good health. There was no family history of dermatologic disorders or toxic exposure, and the parents were not consanguineous. Large bubble-like and exfoliated rashes were observed on her body, mainly distributed in the corners of the eyes, around the mouth, buttocks, and limbs, partially broken and bleeding (Figure 1A–C). Her hair was sparse, soft, and brown. Laboratory investigations, including complete blood count, procalcitonin, liver and kidney function, serum albumin levels, electrolyte, and urine and stool examination, showed normal results. The level of serum zinc was low (0.7 μmol/L; normal range, 9.8–16.8 μmol/L). In addition, the results of microbiological tests showed that the patient had a bacterial skin super infection with *Pseudomonas aeruginosa*. Combined with the clinical manifestations and laboratory examination findings, the patient was diagnosed with AE and zinc deficiency. The rashes improved after being treated with a zinc preparation, fusidic acid cream, recombinant bovine basic fibroblast growth factor gel, and piperacillin sulbactam sodium (Figure 2A, B).

## Results

**Figure 3:**
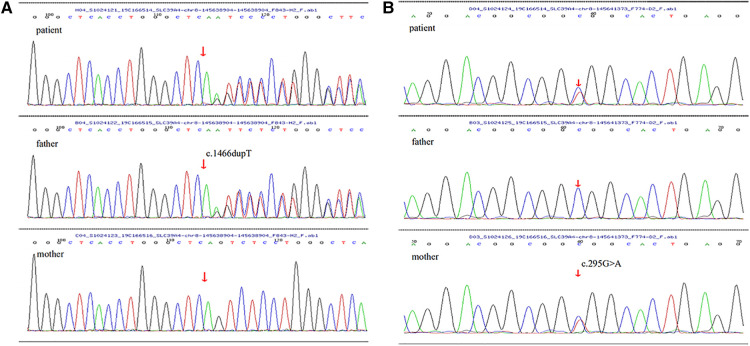
(**A,B**) The arrow of *SLC39A4* gene sequencing maps of the child and her parents showed mutations: (**A**) there was a novel frameshift mutation (c.1466dupT) in exon 9, originated from her father, leading to amino acid changes (p.S490Efs*155). (**B**) There was a missense mutation (c. 295G > A) in exon 2, inherited from her mother, leading to a substitution of the 99th amino acid alanine with threonine (p.A99T).

After obtaining written informed consent from the patient and her parent, the peripheral blood samples of the child and her parent were collected to do the related gene detection. The result showed a novel heterozygous mutation in the *SLC39A4* gene c.1466dupT in exon 9 and c. 295G > A in exon 2, which lead to frameshift mutation (p.S490Efs*155) and results in a change from alanine to threonine at amino acid position 99(p.A99T). Genetic testing of family members revealed that the patient’s father was found to only carry heterozygous c.1466dupT (p.S490Efs*155) mutation, while her mother carried heterozygous 295G > A (p.A99T) mutation (Figures 3A,B).

## Discussion

AE is a very rare autosomal recessive disorder with an incidence of 1 per 500,000 children without apparent predilection for race or sex (3). At present, it has been found that the *SLC39A4* gene located on chromosome 8q24.3 is the pathogenic gene of AE. The gene contains 12 exons and 11 introns, which is about 4.7 kb in length, and encodes a zinc-specific transporter (Zip4) (2).

Zinc is one of the most important trace elements in the human body, as approximately 10% of the proteins in the human proteome are zinc dependent. The functions of zinc have been divided into three categories: catalysis; structural components; and regulation. It is widely involved in physiological processes such as body growth, intellectual development, cell proliferation, substance metabolism, wound healing, and tissue repair (4, 5). Therefore, impaired zinc homeostasis and subsequent zinc deficiency can provoke the onset and progression of a number of diseases, some of which can be a threat to life (5). Zinc deficiency accounts for 4% of the morbidity and mortality of children aged 6 months to 5 years worldwide (6). Zinc deficiency is associated with severe anemia, growth restriction, hypogonadism, skin abnormalities, and mental malaise, and can also cause a wide range of symptoms, including persistent diarrhea, alopecia, taste disorders, immune insufficiency, impairment of wound healing, chronic inflammation, and neuropsychiatric changes (5). A laboratory examination showed that there was also a decrease in serum zinc levels in this case, and the main clinical manifestations of the patient were recurrent skin rash and alopecia.

ZIP4, a zinc transporter encoded by *SLC39A4* gene, is responsible for the transport of zinc to the intestinal epithelial cells of the duodenum and jejunum, from the intracellular vesicles to the cytoplasm (7). Pathogenic mutations in AE have been proved to result in defects in zinc responsive trafficking to theplasma membrane, reduced zinc uptake activity , or defects in processing, in which the extracellular amino-terminal domain of ZIP4 undergoes proteolytic cleavage during extended periods of zinc deficiency, which can result in a decrease in intestinal zinc uptake, resulting in a decrease in intracellular, serum, or plasma zinc levels (6, 8). Individuals with AE have severe zinc deficiency derived from a defect of zinc absorption in the duodenum and jejunum (7).

The clinical symptoms of AE usually appear within several weeks or months after birth, with the highest incidence around weaning age, and they vary with age (9, 10). AE is characterized by periorificial dermatitis, alopecia, and diarrhea. These three symptoms simultaneously occur in only 20% of patients. The severity of the skin lesions is variable. The disease begins with symmetrical erythematous, squamous or eczematous lesions, sometimes vesiculobullous or pustular lesions, located around the perioral, anogenital, and acral areas. In severe cases, skin damage spreads to other periorificial areas of the face (eyes, nose, and ears), neck, lower abdomen, back, inguinal region, and thighs. In addition, some skin rashes can also become erosive or similar to a psoriatic rash (4). Other mucous and cutaneous signs include diffuse alopecia, loss of eyelashes and eyebrows, glossitis, gingivitis, stomatitis, onychodystrophy, onycholysis, and pachyonychia (11). The degree of diarrhea is consistent with the degree of zinc deficiency. From no diarrhea symptoms to intermittent and even persistent diarrhea, the degree of zinc deficiency is further aggravated (4). Advanced symptoms of AE may include neuropsychiatric disorders, hypogonadism, growth restriction, and immune system dysfunction. Untreated patients with AE may eventually end up with multiple organ failure and even death (10). In addition, the skin lesions of patients with AE can develop secondary bacterial infection, which were mostly caused by *Candida albicans* and Gram-positive bacteria (4)*.* Our patient had bacterial skin super infections with *P. aeruginosa* and was given treatment with antibiotics according to susceptibility.

Zinc deficiency can be divided into hereditary zinc deficiency (AE) and acquired zinc deficiency. AE is a genetic disease caused by *SLC39A4* gene mutation, and acquired zinc deficiency is a nutritional disease caused by temporary zinc supplementation or inadequate absorption, and its onset is unrelated to the *SLC39A4* gene, so it often does not recur after treatment with zinc supplementation. The early symptoms are the same in both AE and zinc deficiency, including dermatitis, diarrhea, and alopecia. Therefore, *SLC39A4* gene screening is an important way of distinguishing these two diseases.

The diagnosis of AE is based on clinical symptoms and improvement after zinc supplementation. Low levels of plasma/serum zinc can further support the diagnosis, and the detection of a pathogenic mutation in the *SLC39A4* gene can finally diagnose AE (12, 13). A low level of serum alkaline phosphatase, a zinc-dependent metalloenzyme, may support the diagnosis of AE (14). However, it has been reported that some patients with AE presented only with a typical skin rash and a normal level of serum zinc. The reason may be that they had been taking an oral zinc supplement intermittently or that one of the *SLC39A4* alleles in the compound heterozygous mutation barely affected the transport of zinc (9, 15).

So far, more than 52 mutations have been reported in patients with AE, including missense mutations, nonsense mutations, frameshift mutations, splice site mutations, and so on (16). According to the literature (10), the common high-frequency mutations of AE are located in exons 9, 3, and 5. Missense mutations account for 71.43% of the gene phenotype. The mutation of exon 9 can occur in men and women and has an average onset age of 15.86 ± 9.21 months. The clinical manifestations are mainly skin and mucosal damage. In our case, AE began at the age of 1 year, with a typical rash and alopecia, the serum zinc level decreased, and the rash improved after zinc supplementation. Based on the gene detection results, the diagnosis of AE was confirmed. The results showed that the gene test of the patient was compound heterozygous for a novel frameshift mutation (c.1466dupT) in exon 9 and a missense mutation (c. 295G > A) in exon 2. Among the two mutations, c.1466dupT was originated from the patient's father, leading to an amino acid change (p.S490Efs*155), and c. 295G > A was inherited from her mother, leading to a substitution of the 99th amino acid alan It can be seen that the above two gene mutations alone may not cause disease, but both appearing at the same time can affect the function of zinc transporters and lead to zinc deficiency and related clinical manifestations.

Once AE is diagnosed, zinc supplementation is needed in time. Unlike in acquired zinc deficiency, AE requires lifelong zinc substitution where discontinuation leads to relapse after about 2 weeks (17). Oral zinc supplementation can produce a significant and rapid therapeutic response in the treatment of AE, but there is no clear consensus or suggestion on the type, dose, and duration of zinc supplementation. Zinc sulfate, zinc acetate, zinc oxide, zinc chloride, and zinc gluconate are recommended by the Food and Drug Administration (FDA) in the United States, and among them, zinc sulfate is the best tolerated (4). However, Kilic et al. (18) reported a patient with AE who was resistant to high-dose zinc sulphate therapy but responded to zinc gluconate treatment. Another article also emphasizes the importance of sustained high-dose zinc supplementation in cases of AE (19). At present, most authors recommend an elemental zinc lifelong dose of 3 mg/kg/day, while some authors advocate the regular supplement of 1–2 mg/kg zinc every day and increase the dose (5–10 mg/kg) when the condition of AE become worse. Whether patients with AE need higher doses during infection, stress, or adolescence needs further research (20). After confirming the diagnosis of our patient, she was treated with a zinc sulfate preparation of 3 mg/kg every day. After 2 weeks, the rash improved. During the six-month follow-up, the rash had not recurred, there was no alopecia, and the level of serum zinc was higher than before.

We found that both c.1466dupT frameshift mutation and c. 295G > A (p.A99T) missense mutation in *SLC39A4* gene existed in our patient. Even though a new compound heterozygous mutation was found in this childhood case, its functions have been not confirmed. This compound heterozygous mutation type has not been reported before, which may enrich the mutation types of the *SLC39A4* gene, and is helpful for pediatricians to understand the relationship between genotype and phenotype in patients with AE. At the same time, gene detection should be carried out as far as possible in children considering the possibility of AE, which is helpful for the diagnosis of AE, and determines whether the child needs long-term zinc supplement treatment, so as to reduce the damage of the many aspects of long-term zinc deficiency to children.

## Data Availability

The datasets for this article are not publicly available due to concerns regarding participant/patient anonymity. Requests to access the datasets should be directed to the corresponding author.
